# Updated distribution maps of predominant *Culex* mosquitoes across the Americas

**DOI:** 10.1186/s13071-021-05051-3

**Published:** 2021-10-23

**Authors:** Morgan E. Gorris, Andrew W. Bartlow, Seth D. Temple, Daniel Romero-Alvarez, Deborah P. Shutt, Jeanne M. Fair, Kimberly A. Kaufeld, Sara Y. Del Valle, Carrie A. Manore

**Affiliations:** 1 Information Systems and Modeling, Los Alamos National Laboratory, Los Alamos, NM USA; 2 Biosecurity and Public Health, Los Alamos National Laboratory, Los Alamos, NM USA; 3 Statistical Sciences, Los Alamos National Laboratory, Los Alamos, NM USA; 4 Department of Statistics, University of Washington, Seattle, WA USA; 5 Biodiversity Institute and Department of Ecology and Evolutionary Biology, University of Kansas, Lawrence, KS USA; 6 OneHealth Research Group, Facultad de Medicina, Universidad de las Américas, Quito, Ecuador

**Keywords:** Mosquitoes, Niche model, Species distribution, Vectors, Mosquito-borne disease, Infectious disease

## Abstract

**Background:**

Estimates of the geographical distribution of *Culex* mosquitoes in the Americas have been limited to state and provincial levels in the United States and Canada and based on data from the 1980s. Since these estimates were made, there have been many more documented observations of mosquitoes and new methods have been developed for species distribution modeling. Moreover, mosquito distributions are affected by environmental conditions, which have changed since the 1980s. This calls for updated estimates of these distributions to understand the risk of emerging and re-emerging mosquito-borne diseases.

**Methods:**

We used contemporary mosquito data, environmental drivers, and a machine learning ecological niche model to create updated estimates of the geographical range of seven predominant *Culex* species across North America and South America: *Culex erraticus*, *Culex nigripalpus*, *Culex pipiens*, *Culex quinquefasciatus*, *Culex restuans*, *Culex salinarius*, and *Culex tarsalis*.

**Results:**

We found that *Culex* mosquito species differ in their geographical range. Each *Culex* species is sensitive to both natural and human-influenced environmental factors, especially climate and land cover type. Some prefer urban environments instead of rural ones, and some are limited to tropical or humid areas. Many are found throughout the Central Plains of the USA.

**Conclusions:**

Our updated contemporary *Culex* distribution maps may be used to assess mosquito-borne disease risk. It is critical to understand the current geographical distributions of these important disease vectors and the key environmental predictors structuring their distributions not only to assess current risk, but also to understand how they will respond to climate change. Since the environmental predictors structuring the geographical distribution of mosquito species varied, we hypothesize that each species may have a different response to climate change.

**Graphical abstract:**

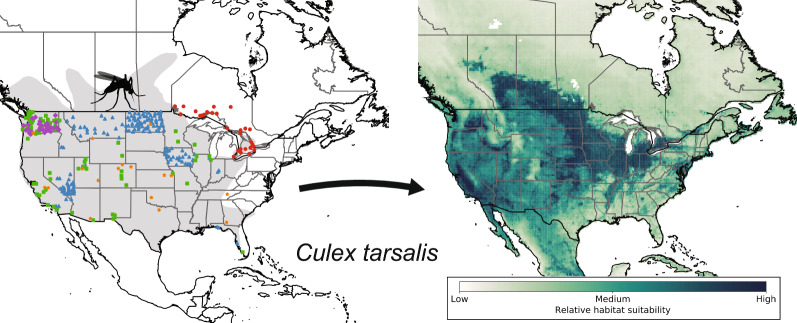

**Supplementary Information:**

The online version contains supplementary material available at 10.1186/s13071-021-05051-3.

## Background

*Culex* mosquitoes, which frequently take blood meals from animals—including humans, are important transmitters of emerging and re-emerging pathogens [1–4]. These mosquitoes are often found in temperate climate zones and are known to be vectors for several arboviruses, including West Nile virus (WNV), St. Louis encephalitis virus (SLEV), eastern equine encephalitis virus (EEEV), and western equine encephalitis virus (WEEV) (Table 1). For example, WNV is the largest cause of mosquito-borne disease in humans in the United States and is maintained in nature in a wild bird-mosquito life-cycle involving a variety of avian hosts and several *Culex* species. *Culex nigripalpus*, *Culex pipiens*, *Culex quinquefasciatus*, and *Culex tarsalis* are highly efficient in maintaining and transmitting WNV [2, 3, 5, 6]. Some species transmit parasites as well, such as filarial nematodes and the *Plasmodium* parasites that cause avian malaria (Table 1).

**Table 1:** Summary of the pathogens that are vectored by the seven focal *Culex* species and the general regions in which these species are found

Species	Pathogen	General region	References
*Culex erraticus*	EEEV, Venezuelan equine encephalitis virus, WNV, Zika virus	Southeastern USA, Midwestern USA, Mexico, South America	[92–95]
*Culex nigripalpus*	EEEV, SLEV, WNV, Zika virus, dog heartworm, avian malaria	Southeastern USA, Mexico, South America	[4, 96–98]
*Culex pipiens*	SLEV, WNV, Zika virus, filarial worms, avian malaria	Mexico, Canada, Midwestern USA, northeast USA	[3, 96, 99]
*Culex quinquefasciatus*	SLEV, WEEV, WNV, Zika virus, lymphatic filariasis	Southern USA, Mexico, South America	[24, 98, 100–102]
*Culex restuans*	SLEV, WNV	Canada, Mexico, eastern USA	[3, 98, 99]
*Culex salinarius*	SLEV, WEEV, WNV	Midwestern USA, northeastern USA, southeastern USA	[98, 99]
*Culex tarsalis*	SLEV, WEEV, WNV, Zika virus	Mexico; west of the Mississippi, USA; southeastern USA	[96, 98, 103]

* EEEV* Eastern equine encephalitis virus, *WNV* West Nile virus,* SLEV* St. Louis encephalitis virus,* WEEV* western equine encephalitis virus

Disease outbreaks facilitated by *Culex* species are increasing in frequency, and these mosquitoes will continue to be important vectors for emergent and re-emerging diseases [7–9]. For example, outbreaks of EEEV occurred in multiple states within the USA in summer 2019, which resulted in 34 infections and 11 deaths [10]. Spillover events of EEEV into dead end hosts, such as humans and horses, occur unpredictably and are mediated by *Culex* mosquitoes [11]. Outbreaks of EEEV and similar diseases are difficult to predict because of the complex interactions between reservoir hosts, environmental conditions, human activity, and mosquito distributions [12]. Understanding the spatial distribution of *Culex* species that are important for the transmission of EEEV and other diseases can provide insight on areas prone to disease risk.

Our current understanding of the geographical distributions of *Culex* species in North America and South America is mainly limited to studies carried out at state and provincial levels in the USA and Canada that were published in the early 1980s [13]. Though the distribution maps have been updated recently with new species and additional presence data, the estimates of the spatial extent of *Culex* species have generally remained the same [14]. Furthermore, climate and environmental conditions have continued to change in the years since these updates, including further warming of surface air temperatures and shifts in precipitation [15].

Significant changes to climate are occurring around the world, which influence where mosquitoes can survive. Areas are becoming warmer and wetter or drier [15], causing the geographic distributions of mosquitoes to expand, contract, and/or shift in response [12, 16–18]. In the near future, it is estimated that North America, Central America, and South America will experience warmer temperatures and more variable precipitation patterns [19, 20]. There will also likely be an increase in the number of extreme weather events, leading to increased flooding in some areas and long-term droughts in others [19]. Increasing temperatures result in quicker life-cycles of mosquitoes, which allow them to colonize new areas within their temperature limits [21, 22]. Changes to precipitation patterns will promote suitable habitats (e.g., standing water) for them in some areas, but decrease suitability where habitats become drier [21].

There is already evidence that several *Culex* species have experienced recent shifts in their geographical ranges, with some species expanding northward into Canada. For example, *Culex salinarius* and *Culex erraticus* have been consistently trapped in Ontario, Canada in recent years [23]. This finding contradicts the distribution maps produced by Darsie and Ward [14], which show *Cx. salinarius* and *Cx. erraticus* having northernmost ranges in the northern and Midwestern USA states, respectively [14]*. Culex pipiens* and *Cx. quinquefasciatus* are both predicted to spread northward, too [17, 24]*.* In fact, *Cx. pipiens* has already been identified in Canada, and it is likely that this species will undergo further range expansion northward [17].

In parallel to expanding mosquito species distributions, several mosquito-borne viruses are expanding as well, impacting novel and susceptible human populations [9, 18, 25]. For example, since 2014, specific strains of SLEV thought to be restricted to Argentina have been reported in Arizona and California [9, 26]. There has also been an increased risk for WNV in Canada, both in urban and rural areas [27]. Knowing which mosquito species and their associated pathogens are present in an area may assist physicians in providing accurate disease diagnoses and timely, appropriate medical treatments, as well as help mosquito control districts identify and control these species [28].

Recent developments in mathematical ecology offer statistical methods to model geographic ranges for organisms when presence records are available. One such method is Maxent [29, 30]. Maxent estimates occurrence intensities by relating species presence data and background locations to environmental predictors in the context of a generalized linear model [31]. It aims to learn the environmental conditions that are suitable for species presence based on the environmental conditions at the recorded presence data points. Because systematically sampling large areas for species presence is difficult, this statistical method provides a tractable and practical way to determine species distributions by making efficient use of species presence data and remotely sensed environmental covariates. This approach also affords information to researchers and public officials because it proposes environmental conditions important to the distribution of the species.

The goal of our study was to use contemporary mosquito and environmental data to update species distribution maps of seven *Culex* species found in North America and South America: *Culex erraticus*, *Culex nigripalpus*, *Culex pipiens*, *Culex quinquefasciatus*, *Culex restuans*, *Culex salinarius*, and *Culex tarsalis*. To achieve this, we first surveyed the literature for articles describing environmental conditions that may be important for these seven *Culex* species. This literature survey highlighted the variables that promote or inhibit *Culex* species’ persistence and provided information on the geographical locations where these species reside. We gathered environmental datasets for use in model development, such as temperature, humidity, and land cover. Then, we used mosquito presence data from several data repositories and the environmental datasets in Maxent to estimate the contemporary distribution of these seven *Culex* species, examining the environmental variables important for structuring the patterns of habitat suitability. Our contemporary *Culex* distribution maps may be used to assess mosquito-borne disease risk and forecast future threats from emerging diseases carried by these *Culex* species.

## Methods

### Mosquito presence data

We focused our analyses on seven predominant *Culex* species found in North America and South America: *Cx. erraticus*, *Cx. nigripalpus*, *Cx. pipiens*, *Cx. quinquefasciatus*, *Cx. restuans*, *Cx. salinarius*, and *Cx. tarsalis*. We chose these species because of the number of diseases they can transmit, the extent of their presence, and the data available. We gathered mosquito presence data from 1990 to 2020 from three data repositories: VectorBase [32], VectorMap (http://vectormap.si.edu; collected 22 September 2020), and the National Ecological Observatory Network ([33]; collected 4 May 2020), and from surveys done by two public health departments—Public Health of Ontario (2002–2017), and Washington State Department of Health (2008–2014). We included observations that had two decimal point precision (~ 1 km) in their latitude and longitude. To account for the clustering of occurrence points and sampling bias, but also maintain the necessary environmental gradient to capture a reliable signal of habitat suitability, we filtered our mosquito presence data using the package spThin in R using a 30-km radial buffer, so that only one species presence point was used within that buffer area [34, 35]. After filtering, we had between 79 and 300 presence data points per species (Additional file 1: Table S1). As a sensitivity analysis during Maxent model development, we also tried 50-, 75-, and 100-km radial buffers, and the results remained stable (data not shown).

### Climate and environmental data

We reviewed the literature on the seven *Culex* species to find articles describing important climate and environmental drivers for mosquito presence. Using our literature review as a basis for variable selection, we gathered climate and environmental data that we found to be relevant for providing mosquito habitat and supporting the mosquito life-cycle. We used data from two different sources: MERRAclim [36] and EarthEnv [37–39]. These data sources provide remotely sensed variables relating to climate, land cover, terrain, and biodiversity at resolutions of approximately 1 and 5 km.

We used the most contemporary available MERRAclim data, which was averaged from 2000 to 2009 [36]. We included five measures of temperature: annual mean temperature, mean diurnal range in temperature, the maximum temperature of the warmest month, the minimum temperature of the coldest month, and the annual range in temperature. We included three measures of atmospheric moisture: annual mean specific humidity, specific humidity of the most humid month, and specific humidity of the least humid month. Each variable was available at 2.5-arcmin (~ 4 km) resolution.

Each EarthEnv dataset spanned its own time frame based on the product. We included one measure of habitat heterogeneity derived from vegetation, called the evenness of the enhanced vegetation index (EVI), which is bounded from zero to one. This dataset was available at 30-arcsec (~ 1 km) resolution and based on the Moderate Resolution Imaging Spectroradiometer (MODIS) EVI product (MOD13Q1 version 5; 250-m resolution) from 2001 to 2005 [39]. We included 12 different land cover classes derived from multiple datasets [GlobCover (2005–2006; v2.2), the MODIS land cover product (MCD12Q1; v051), GLC2000 (global product; v1.1), and DISCover (GLCC; v2)]. These classifications are available at 30-arcsec (~ 1 km) resolution: evergreen/deciduous needleleaf trees, evergreen broadleaf trees, deciduous broadleaf trees, mixed/other trees, shrubs, herbaceous vegetation, cultivated and managed vegetation, regularly flooded vegetation, urban/built-up, snow/ice, barren, and open water [38]. Lastly, we included four measures of topography based upon the global 250-m GMTED2010 and near-global 90-m SRTM4.1dev products: elevation, slope, roughness, and a terrain ruggedness index [37].

We aggregated each dataset from its native resolution to match the 30-km resolution of our filtered presence data. Matching these resolutions ensures that only one presence data point falls within a single environmental grid. As a result, Maxent analyzes the occurrence record and the environmental grid as a coordinate pair. We visually inspected this aggregated data to verify that coarsening did not noticeably alter the geographic pattern of the environmental variables. We included all the environmental variables in each model in order to infer the contribution of different predictors for each species.

### Maxent modeling

We used the machine learning, maximum entropy model Maxent (maxnet package, version 0.1.2, based on Maxent version 3.4.0; [29, 30]) to create species distribution maps of the seven predominant *Culex* species. We used the 30-km filtered presence data and 30-km climate and environmental data as input to the model. To select the environmental training area for Maxent (i.e., the study area M as defined by Soberon and Peterson [40]), we created a buffer distance for each species based on its presence data and our assumption that mosquito species can readily disperse given their short life-cycles and flight ranges [41–43]. We calculated the buffer length by first computing the centroid of each species’ presence data points and then computing the median distance from each presence point to the centroid [44]. We applied this median distance as the radius for a buffer around each presence point. This procedure creates an environmental training area unique to each species (Additional file 1: Figs. S1, S2). From this environmental training area, we initiated Maxent to randomly sample 10,000 background points and trained the models using cross-validation with ten random k-folds of the presence data points. We considered three different feature classes to model relationships between environmental predictors and presence data: linear (L); linear and quadratic (LQ); and linear, quadratic, and hinge (LQH) [45]. We also tested a suite of regularization parameters for model fit, including 0.5, 1, 2, 5, 10, 20. Larger regularization parameters encourage models with fewer covariates, lowering overfitting [46].

We selected a best model for each species based on criteria calculated from the ENMevaluate package ([47]; version 0.3.1). Maxent practitioners commonly make model selections based on the corrected Akaike information criterion (AICc), but this approach suffers from poor geographic prediction accuracy [48]. On the other hand, models built by optimizing prediction accuracy may fail to detect environmental features of biological importance [49]. In acknowledgment of these shortcomings, we implemented a custom procedure to select our final models. First, we subsetted the models to only consider the half of the models with the lowest absolute bias in omission rates. For the omission rate, we used the percent of testing presences that had a predicted suitability of less than the 10th quantile of the predicted suitability among the training presences (avg.test.or10pct; [50]). Second, we further subsetted the models to include those that passed a threshold based on the difference between the training and testing area under the curve (AUC) averaged across the ten k-fold bins (avg.diff.AUC). That threshold was set as the median average AUC difference among the fitted models. After these two steps, we selected the model with the lowest AICc. This ad hoc procedure balances prediction accuracy in geographic space and model explainability in ecological space.

After selecting the best Maxent model for each species (i.e., feature classes and regularization parameters; Additional file 1: Table S2), we obtained ten model output replicates to assess the variability in our predictions [35, 51]. For the ten replicates, we bootstrapped our mosquito presence data to use 80% of the available presence data. We used the difference between the maximum and minimum habitat suitability output among the ten bootstrapped replicates (i.e., the range) to show areas of higher or lower uncertainty in our models [51, 52].

We were able to analyze the role of each environmental variable in the Maxent models using the mean permutation importance and mean percent variable contribution calculated across the ten bootstrapped models [53]. The permutation importance describes the most important factors in determining the habitat suitability. For each environmental variable in turn, its values are randomly permuted, the model performance is recalculated based on the permuted training data, and the change in model performance is recorded. These differences are then normalized to percentages, where a higher percentage means the model depends heavily on that variable [54]. Maxent also calculates percent contributions from each variable, but these are heuristically defined based on the path the algorithm takes to a locally optimal solution. Although they should be interpreted with caution, together the percent variable contribution and variable permutation importance highlight important environmental predictors.

After training the Maxent models with species-specific background environments (Additional file 1: Figs. S1, S2), we extrapolated each bootstrapped model to create mean species distribution maps across the continents in which we expected the mosquito species to occur. We analyzed *Cx. pipiens*, *Cx. tarsalis*, *Cx. salinarius*, and *Cx. restuans* for North America and *Cx. erraticus*, *Cx. quinquefasciatus*, and *Cx. nigripalpus* for North America and South America. For a large number of background points, a Maxent model can be viewed as a Poisson regression model and its outputs can be interpreted as occurrence intensities from a spatial point process [31]. This equivalency motivates the complementary log–log transformation applied to these model intensities as probabilities of presence, subject to some assumptions about the sampling scheme [30]. We used these transformed model outputs because they are normalized to the [0, 1] range, but we caution against the aforementioned interpretation; instead, we describe the outputs as “relative habitat suitability” with “low” suitability marked at 0, “medium” suitability marked at 0.5, and “high” suitability marked at 1. These measures of relative suitability are unique for each *Culex* species based on the unique Maxent model development.

We also created maps to identify any areas with novel climate or environmental conditions outside of the background environmental training data, so that decisions can be weighed appropriately where the model is extrapolated. To do this, we calculated the species-specific ranges of background environmental data variables and highlighted any geographical areas on the map in which at least one variable was outside of these ranges.

## Results

### Environmental drivers of *Culex* mosquito distributions

We surveyed the literature to identify the geographic region in which each *Culex* species has been documented and which diseases each species may transmit (Table 1). While all seven species have been documented in North America, only three of the seven have been documented in South America.

We also identified environmental drivers that may be important in structuring the geographical distribution of the seven *Culex* species of interest (Additional file 1: Table S3) [16, 17, 42, 55–85]. Each *Culex* species is sensitive to both natural and human-influenced environmental factors, especially land cover and vegetation type. For example, some *Culex* species were less likely to be found in urban areas, such as *Cx. tarsalis* [55] and *Cx. nigripalpus* [56], whereas others were more likely to be found in these, such as *Cx. pipiens* [55, 57, 58] and *Cx. quinquefasciatus* [59]. To account for these differences, we used 12 different land cover types as predictors in our species distribution models, including an “urban/built up” layer. We found evidence that temperature and measures of environmental moisture are important drivers for most *Culex* species (Additional file 1: Table S3). For this reason, we incorporated both temperature and humidity variables as predictors in our models.

### Species distribution maps of* Culex* mosquitoes

**Fig. 1:**
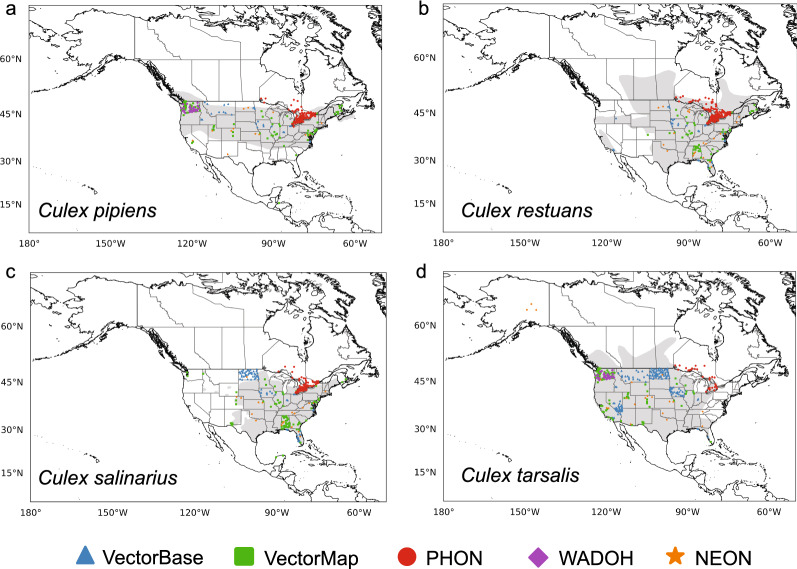
The presence data points used in Maxent model development from several data repositories for **a** *Culex pipiens*, **b** *Culex restuans*, **c** *Culex salinarius*, and **d** *Culex tarsalis*. The estimated distribution of each species within the USA and Canada is shaded in* gray* (from Darsie et al. [14]).* PHON* Public Health of Ontario,* WADOH* Washington State Department of Health,* NEON* National Ecological Observatory Network

**Fig. 2:**
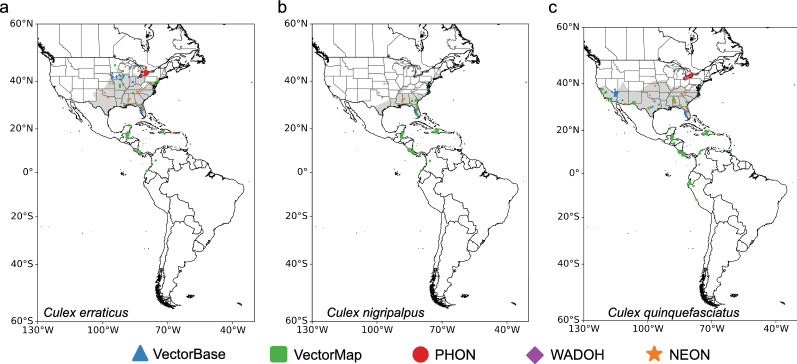
The presence data points used in Maxent model development from several data repositories for **a** *Culex erraticus*, **b** *Culex nigripalpus*, and **c** *Culex quinquefasciatus*. The estimated distribution of each species within the USA and Canada is shaded in* gray* (from Darsie et al. [14]). For abbreviations, see Fig. 1

We gathered contemporary mosquito presence data for the *Culex* mosquito species in North America (Fig. 1) and North America and South America (Fig. 2) for use in the Maxent models. We tried to find presence data to cover the hypothesized geographical range within the USA and Canada highlighted by Darsie and Ward [14]. Though the study of Darsie and Ward [14] was limited to estimating the range within the USA and Canada, many of the presence data points fell within the estimated range. All seven species also had contemporary presence points further north than depicted by Darsie and Ward [14].

**Fig. 3:**
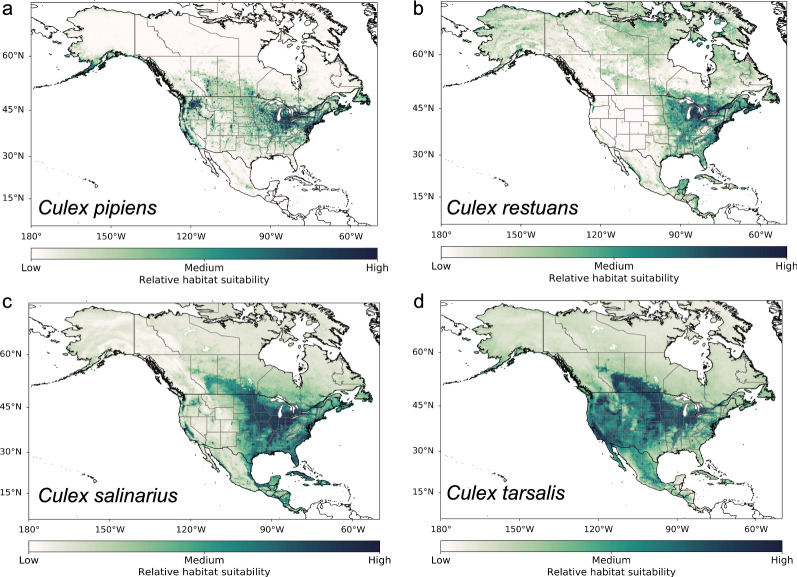
Mean geographical distribution maps averaged across the ten bootstrapped models for predominant *Culex* species in North America, including **a** *Culex pipiens*, **b** *Culex restuans*, **c** *Culex salinarius*, and **d** *Culex tarsalis*. The relative habitat suitability is unique to each species based on the Maxent model development

**Fig. 4:**
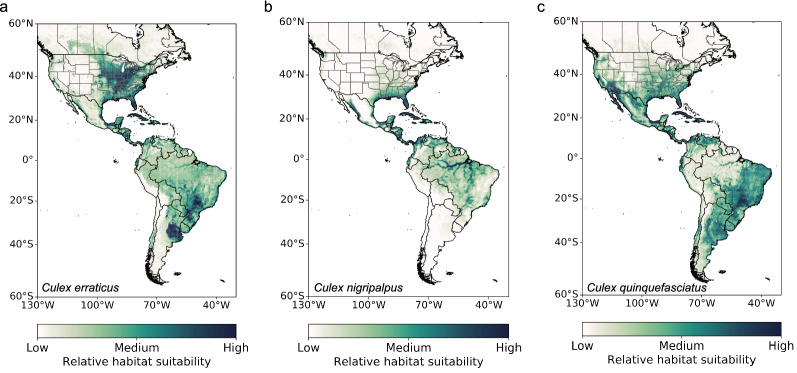
Mean geographical distribution maps averaged across the ten bootstrapped models for predominant *Culex* species in North America and South America, including **a** *Culex erraticus*, **b** *Culex nigripalpus*, and **c** *Culex quinquefasciatus*. The relative habitat suitability is unique to each species based on the Maxent model development

The feature classes and regularization parameters varied among the species for the best Maxent model configuration (Additional file 1: Table S2). We also reported measures of prediction accuracy in geographic space (omission rates and AUC) and model explainability (AICc). After selecting the final model configuration and running the bootstrapped models, the resulting mean species distribution maps showed variability in the species ranges between all seven species, both in North America (Fig. 3) and North America and South America (Fig. 4). In North America, *Cx. pipiens* had the most disparate habitat suitability surrounding urban areas, whereas *Cx. tarsalis* had the most widespread suitability across the temperate and subtropical regions of North America, throughout both urban and rural areas. The geographical ranges of *Cx. restuans* and *Cx. salinarius* were more limited to the eastern and Midwestern USA and the Central Plains of Canada, with the range for *Cx. salinarius* dropping further south into Central America and the Caribbean. In North America and South America, *Cx. erraticus* and *Cx. quinquefasciatus* had a larger range of habitat suitability compared to *Cx. nigripalpus*, which was more limited to the tropics and subtropics. In North America, *Cx. erraticus* showed a similar pattern of habitat suitability to *Cx. salinarius*.

The geographical range of *Cx. pipiens* was largely driven by the urban/built-up land cover variable, which was also an important driver for the distributions of *Cx. salinarius*, *Cx. tarsalis*, and *Cx. quinquefasciatus* (Table 2; Additional file 1: Table S4). Measures of temperature were important drivers for five of the *Culex* species, while not as important for characterizing the distributions of *Cx. nigripalpus* and *Cx. salinarius*. The distributions of all species besides *Cx. quinquefasciatus* were driven by measures of specific humidity. Cultivated and managed vegetation was another land cover variable that was important for several species, including *Cx. erraticus*, *Cx. pipiens*, *Cx. salinarius*, and *Cx. tarsalis*—all of which are noted as predominant mosquitoes in the agricultural plains of the USA. Taken in combination, the percent variable contribution (Additional file 1: Table S4) and variable permutation importance (Table 2) highlight particularly important variables in structuring habitat suitability for different *Culex* mosquito species.

**Table 2:** Percent permutation importance of each environmental variable from Maxent models for each *Culex* species

Environmental variable	*Culex pipiens*	*Culex restuans*	*Culex salinarius*	*Culex tarsalis*	*Culex erraticus*	*Culex nigripalpus*	*Culex quinquefasciatus*
Climate							
Annual mean temp.	26.7	4.8	6.5	6.6	0.0	1.3	2.9
Temp. annual range	0.3	5.4	0.0	0.0	1.6	0.0	0.0
Mean diurnal temp. range	1.1	1.8	0.0	0.0	0.0	0.0	18.4
Maximum temp. in the warmest month	0.0	9.0	0.0	9.5	0.0	0.3	8.9
Minimum temp. in the coldest month	12.5	0.4	2.3	8.4	18.7	0.0	15.1
Annual mean specific humidity	3.3	13.5	12.6	3.8	0.0	0.0	0.0
Specific humidity in the most humid month	10.8	1.1	1.7	30.0	18.4	60.3	2.8
Specific humidity in the least humid month	0.5	14.6	0.0	0.0	3.4	7.4	1.4
Land cover							
Evergreen/deciduous needleleaf trees	0.1	9.2	0.1	0.2	0.4	3.3	1.0
Evergreen broadleaf trees	0.0	0.7	0.0	0.0	1.1	0.4	13.3
Deciduous broadleaf trees	0.2	2.0	0.3	0.8	0.1	1.5	0.4
Mixed/other trees	0.3	1.8	0.0	0.0	12.1	0.5	1.8
Shrubs	0.0	1.9	2.4	0.0	2.1	0.7	4.9
Herbaceous vegetation	0.1	12.1	0.8	0.1	10.1	0.5	2.0
Cultivated and managed vegetation	12.3	2.0	30.2	27.2	13.8	1.6	3.2
Regularly flooded vegetation	0.0	3.3	0.0	0.0	0.0	0.0	0.0
Urban/built-up	25.6	1.2	21.9	12.4	5.7	0.8	14.1
Snow/ice	0.0	0.0	0.0	0.5	0.0	< 0.1	0.0
Barren	0.0	< 0.1	0.0	0.0	0.0	0.0	0.5
Open water	0.4	1.6	0.9	0.0	0.4	0.2	0.6
Habitat							
Evenness of EVI	0.1	7.5	0.0	0.0	3.7	2.9	0.0
Topography							
Elevation	5.7	0.7	11.9	0.5	8.4	9.6	5.9
Roughness index	0.0	0.0	0.0	0.0	0.0	0.0	0.0
Slope	0.0	5.3	8.4	0.0	0.0	3.1	2.5
Terrain ruggedness index	0.0	0.0	0.0	0.0	0.0	5.5	0.1

*Temp*. Temperature, *EVI* enhanced vegetation index

Although each model was developed from a unique set of background environmental training data, we extrapolated the models to all of North America and/or South America. By doing so, in some areas the models are applied to novel climate or environmental conditions. We created maps to identify these areas (Additional file 1: Figs. S3, S4). If using the map for decision support, these areas should be addressed with caution. These areas mostly include northern boreal and arctic regions, some tropical regions, areas with high elevation, or areas with exceptionally dry conditions.

The range in habitat suitability across the bootstrapped models was low for *Cx. pipiens*, *Cx. salinarius*, and *Cx. tarsalis*, displaying confidence in the model output across North America (Additional file 1: Fig. S5). The suitability range for *Cx. restuans* was highly variable in Central America, so caution should be used when interpreting the results for that region. This area was outside the background environmental data used to train the Maxent model for *Cx. restuans* and coincides with areas where the model was applied to novel environmental conditions (Additional file 1: Figs. S1, S4). The ranges for *Cx. erraticus*, *Cx. nigripalpus*, and *Cx. quinquefasciatus* all have relatively higher levels of uncertainty, especially across South America (Additional file 1: Fig. S6).

## Discussion

We used contemporary mosquito data, environmental drivers, and an ecological niche model to create updated estimates of the geographical range of seven predominant *Culex* species in North America and South America. We found that the geographical range varied across the seven species, but measures of temperature, humidity, urban/built-up land class, and cultivated and managed vegetation were especially important environmental drivers structuring the spatial distribution of these species. The geographical distributions highlighted known preferences of the different species; for example, the range of *Cx. pipiens* indicated that urban environments were more favorable for this species, whereas the range of *Cx. tarsalis* indicated that the environments throughout the Great Plains of the USA, the Central Valley of California, and other agricultural areas, were most favorable for this species. Urban/build-up land cover was an important variable for structuring the spatial distribution of *Cx. pipiens*, *Cx. quinquefasciatus*, *Cx. salinarius*, and *Cx. tarsalis*; however, the complexities of the Maxent model structure make it challenging to identify the relationship between environmental drivers and the presence of these mosquitoes since there are numerous parameters and model coefficients that could be compensating for other similar drivers.

Our *Culex* species distribution maps leverage contemporary mosquito presence data from several sources to update the hypothesized distribution for seven important disease vectors. The majority of these data sources classify which *Culex* species is present by using morphology, which is prone to error [86]. The *Cx. pipiens* species complex consists of several morphologically similar species that are difficult to visually identify [87], which includes *Cx. pipiens* and *Cx. quinquefasciatus* within our study. In fact, some databases allow generic species identification entries of “*Culex pipiens* morphological group”, which we did not include in our Maxent models to reduce uncertainty. However, one of our data sources, VectorBase, includes *Culex* species identified from genomics data, eliminating some of the error from misidentification. Creating a species distribution map using genomics data alone would increase the precision of the estimated geographical ranges; currently, this approach is not feasible given the limited number of presence data from sequencing. Identifying the correct species is important since they can play different roles in disease transmission. For example, for WNV, while *Cx. pipiens* primarily feeds on birds, *Cx. quinquefasciatus* feeds on both birds and mammals, which may act as a bridge to amplify the transmission of WNV between avian and mammalian hosts [88]. Using genomics data can also highlight areas where there are hybrids, which can alter disease transmission dynamics [89]. With so many agencies in the USA collecting mosquito data for both abatement and control measures and scientific research, it would be transformative to have a central data repository for mosquito species and disease incidence data.

Our maps may be suitable for estimating where a species could readily spread if introduced to a new area, targeting potential environmental sampling for a species, or analyzing potential disease pathways in response to an emergent mosquito-borne disease. However, there are several considerations when analyzing the maps. First, the majority of our presence data points were limited to North America, which could potentially generate more uncertainty for the hypothesized distributions in South America. Second, these distribution maps are a measure of relative habitat suitability rather than the true presence of the organism. Other factors, such as interannual climate conditions, land use, and species migration, govern the presence of the organism within the potential distribution [90]. Lastly, due to the complex nature of Maxent models, they may behave in unexpected ways when extrapolated to novel climate conditions. It is important to recognize areas with novel climate conditions relative to the background environmental data; we note that these areas should be flagged as more uncertain when using these maps for applications.

Our distribution maps corroborate niche models recently developed for *Culex* species using different data sources and modeling techniques at other spatial scales. A global study of the distribution of *Cx. quinquefasciatus* using more presence data points in Central America and South America but fewer in the USA and Canada shows a similar pattern of *Cx. quinquefasciatus* limited to the southern part of the USA and suitable habitat throughout Central America and much of South America [24]. However, our map also shows a low level of habitat suitability throughout much of the Amazon rainforest. A study using logistic regression models of *Cx. pipiens* in Canada shows similar results of the geographical range limited to the extreme southeastern portion of Canada, including southern Ontario, Quebec, and Nova Scotia [17]. Our maps are limited by a lower spatial resolution to encompass a greater physical distance, so we cannot capture high resolution nuances in habitat suitability for a species compared to smaller-scale, high resolution studies. For example, our map highlights St. Johns County, Florida, USA as suitable for both *Cx. nigripalpus* and *Cx. quinquefasciatus*. However, a county-level species distribution map using Maxent and seropositive records of sentinel chickens as presence data points shows heterogeneity in suitability of habitats for these mosquito species within the county [60].

Understanding the contemporary distributions of the predominant *Culex* mosquitoes is critical, as their distributions will likely shift in response to climate change. This may expose new communities to mosquito-borne diseases, or result in emerging diseases [12]. Maxent models can also be used to project future mosquito distributions in response to climate change scenarios, pending reliable projections of future environmental and climate conditions (e.g., [24]). Warming temperatures may cause the geographical range of mosquito species to expand further north [17, 24]. However, increasing temperatures throughout the tropics and subtropics may begin to push some *Culex* species above their thermotolerance, causing mortality and making certain habitats or regions unsuitable for them [91]. The exact response to increasing temperatures varies between *Culex* species; for example, *Cx. quinquefasciatus* is likely more adapted to survive higher temperatures, while *Cx. pipiens* may be more sensitive to them [42].

## Conclusions

Understanding the geographical distributions of disease vectors, such as mosquitoes, is critical for understanding disease risk. We created updated distribution maps of *Culex* mosquitoes, which are vectors for numerous diseases, throughout North America and South America using contemporary observation data and a machine learning ecological niche model. Our distribution maps provide insight on the key drivers structuring the spatial distribution of *Culex* mosquitos. Understanding the distribution of vectors is particularly important when a new disease emerges and rapid assessments need to be made for disease mitigation strategies. These estimates can help identify which communities are most at risk based on the primary disease vectors, and provide decision support regarding where to spray pesticides, and/or where to allocate healthcare resources. Further, a baseline understanding of which environmental conditions structure the geographical distributions of vector species is necessary in order to create projections of disease risk in response to climate change. As temperatures warm, precipitation patterns change, and landscapes shift in response to climate change, the geographical distributions of disease vectors, and therefore the diseases they carry, will also change. Since the key environmental drivers and resultant distribution maps for each *Culex* species were different, we hypothesize that each species will show a unique response to climate change.

## Supplementary Information

**Additional file 1: Table S1.** The total number of presence data points for each *Culex* species used in model development after filtering by the 30-km radial buffer. **Table S2.** Final model specifications and performance metrics for each species. **Table S3.** Summary of environmental factors important for each of the seven *Culex* species, which were obtained from the literature review. **Table S4.** Percent environmental variable contribution during Maxent model development for each *Culex* species. **Figure S1.** Maps of the environmental training area unique to each species used for the Maxent models across North America for** a** *Culex pipiens*, **b** *Culex restuans*,** c** *Culex salinarius*, and** d** *Culex tarsalis*. These were created by buffering the data based on the median distance from each presence data point to the centroid of all presence points. Ten thousand background points are randomly sampled from the shaded environmental training area when running Maxent. **Figure S2.** Maps of the environmental training area unique to each species used for the Maxent models across North America and South America for** a** *Culex erraticus*,** b** *Culex nigripalpus*, and** c** *Culex quinquefasciatus*. These were created by buffering the data based on the median distance from each presence data point to the centroid of all presence points. Ten thousand background points are randomly sampled from the shaded environmental training area when running Maxent. **Figure S3.** After extrapolating the Maxent models across North America, areas that are highlighted have novel climate or environmental conditions relative to the background environmental training dataset. This is unique to each species:** a** *Culex pipiens*,** b** *Culex restuans*,** c** *Culex salinarius*, and** d** *Culex tarsalis*. **Figure S4.** After extrapolating the Maxent models across North America and South America, areas that are highlighted have novel climate or environmental conditions relative to the background environmental training dataset. This is unique to each species:** a** *Culex erraticus*,** b** *Culex nigripalpus*, and** c** *Culex quinquefasciatus*. **Figure S5.** Maps of the difference between the maximum and minimum suitability output amongst the ten bootstrapped replicates (i.e., the range) to show areas of high or low uncertainty in our models for species in North America:** a** *Culex pipiens*,** b** *Culex restuans*,** c** *Culex salinarius*, and** d** *Culex tarsalis*. **Figure S6.** Maps of the difference between the maximum and minimum suitability output amongst the ten bootstrapped replicates (i.e., the range) to show areas of high or low uncertainty in our models for species in North America and South America:** a** *Culex erraticus*,** b** *Culex nigripalpus*, and** c** *Culex quinquefasciatus*.

## Data Availability

The habitat suitability values for each mosquito species across the full spatial domains are available as netCDF files at https://github.com/lanl/culexmaxentmodels.
