# Supplementary material for: Updated distribution maps of predominant Culex mosquitoes across the Americas
Source: Parasit Vectors. 2021 Oct 23;14:547. doi: 10.1186/s13071-021-05051-3 (PMC8542338; doi:10.1186/s13071-021-05051-3)

Supplemental information
Additional file 1

Updated distribution maps of predominant Culex mosquitoes across the Americas

Morgan E. Gorris[1], Andrew W. Bartlow[2], Seth D. Temple[3,4], Daniel Romero-Alvarez[1,5,6], Deborah P. Shutt[1], Jeanne M. Fair[2], Kimberly A. Kaufeld[3], Sara Y. Del Valle[1], Carrie A. Manore[1]

[1]Information Systems and Modeling, Los Alamos National Laboratory, Los Alamos, NM, USA
[2]Biosecurity and Public Health, Los Alamos National Laboratory, Los Alamos, NM, USA
[3]Statistical Sciences, Los Alamos National Laboratory, Los Alamos, NM, USA
[4]Department of Statistics, University of Washington, Seattle, WA, USA
[5]Biodiversity Institute and Department of Ecology & Evolutionary Biology, University of Kansas, Lawrence, KS, USA
[6]OneHealth Research Group, Facultad de Medicina, Universidad de las Américas, Quito, Ecuador

Tables:

Table S1. The total number of presence data points for each *Culex* species used in model development, after filtering by the 30 km radial buffer.

***Culex* species**	**VectorBase**	**VectorMap**	**NEON**	**PHON**	**WADOH**	**Total**
Pipiens	27	81	13	96	29	**246**
Restuans	33	51	21	122	0	**227**
Salinarius	74	70	19	66	0	**229**
Tarsalis	150	71	20	28	31	**300**
Erraticus	35	43	18	15	0	**111**
Nigripalpus	20	46	13	0	0	**79**
Quinquefasciatus	45	76	15	10	0	**146**

Note: PHON is the Public Health Department of Ontario and WADOH is the Washington State Department of Health.

Table S2. Final model specifications and performance metrics for each species.

Culex species	Feature classes	λ	Train AUC	Mean Test AUC	Mean Difference AUC	Mean Test OR 10%	Var. Test OR 10%	AICc	ΔAICc	# of parameters
Pipiens	LQH	10	0.92	0.91	0.022	0.121	0.006	4261	43	37
Restuans	LQ	2	0.87	0.86	0.018	0.115	0.007	4033	45	21
Salinarius	LQH	20	0.85	0.83	0.019	0.115	0.004	4309	140	11
Tarsalis	LQH	20	0.85	0.84	0.015	0.099	0.005	5658	300	15
Erraticus	LQ	10	0.87	0.85	0.034	0.136	0.035	2049	20	11
Nigripalpus	L	5	0.89	0.87	0.039	0.129	0.012	1362	12	13
Quinque-fasciatus	LQH	10	0.90	0.88	0.024	0.131	0.011	2754	29	23

Note: The feature classes are denoted as L for linear function, Q for quadratic function, and H for hinge function. Regularization parameters are denoted as "λ", "AUC" is area under the curve, and "OR" is omission rate.

Table S3. Summary of environmental factors important for each of the seven *Culex* species obtained from the literature review

Species	Variable class for distribution modeling	Environmental variables	Relationships between factor and mosquito	Region over which conclusions were made	References
Culex erraticus	Land cover	Agricultural wetlands	Positive	Gulf Coastal Plain of Georgia, USA	[79]
	Land cover	Trees and buttress roots	Encountered more often in hollow trees, and buttress roots	Costa Rica	[80]
	Land cover	Bermuda grass	Higher number of mosquitoes oviposited in traps with Bermuda grass	San Antonio, TX, USA	[81]
	Land cover	Unshaded	Prefers these conditions	Venezuela	[82]
	Climate	Warm	Prefers these conditions	Venezuela	[82]
	Land cover	Vegetated waters in flooded pastures	Prefers these conditions	Venezuela	[82]
	Land cover	Swamp	Prefers these conditions	Venezuela	[82]
	Land cover	Lagoon	Prefers these conditions	Venezuela	[82]
	Land cover	Ground pools	Prefers these conditions	Venezuela	[82]
	Land cover	Open areas	Prefers these conditions	Venezuela	[82]
	Land cover	Eastern red cedar	Prefers these conditions	Oklahoma, USA	[83]
	Land cover	Deciduous woodlands	Prefers these conditions	Oklahoma, USA	[83]
Culex nigripalpus	Climate	Precipitation of driest month	Positively correlated	St. Johns County, FL, USA	[65]
	Climate	Precipitation in wettest month	Negatively correlated	St. Johns County, FL, USA	[65]
	Climate	Temperature seasonality	Negatively correlated	St. Johns County, FL, USA	[65]
	Climate	Mean temperature of coldest quarter	Negatively correlated	St. Johns County, FL, USA	[65]
	Land cover	Urban	Negatively correlated	St. Johns County, FL, USA	[65]
	Climate	Isothermality	Negatively correlated	St. Johns County, FL, USA	[65]

	Climate	Annual precipitation	Negatively correlated	St. Johns County, FL, USA	[65]
	Land cover	Hill shade	Negatively correlated	St. Johns County, FL, USA	[65]
	Land cover	Bermuda grass	Higher number of mosquitoes oviposited	San Antonio, TX, USA	[81]
	Land cover	Mixed vegetation	Negative	Florida, USA	
	Land cover	Roads (paved)	Prefers these conditions	Florida, USA	[84]
	Land cover	Water (ponds)	Prefers these conditions	Florida, USA	[84]
	Climate	High humidity	Favor high humidity	Florida, USA	[85]
	Climate	Wet conditions	Prefers these conditions	Florida, USA	[86]
	Land cover	Standing water	Prefers these conditions	Florida, USA	[86]
	Land cover	Urban areas	Rarely found in urban areas	Florida, USA	[56]
Culex pipiens	Land Cover	Urban areas	Human WNV disease incidence in Northeastern regions was positively associated with urban land covers.	Northeastern USA	[57]
	Climate	Temperature	Female survival decreased significantly between 20 and 24C	Laboratory study	[42]
	Land cover	Urban and suburban areas	Mostly urban and suburban mosquito	Iowa, USA	[55]
	Climate	Temperature (means, mins, maxs, over certain periods, etc.)	Thrive in warm conditions, but not extremely hot conditions	Canada	[17]
	Climate	Precipitation (annual, monthly)	Larva develop in standing water sites	Canada	[17]
	Land cover	Agricultural land	Within 2 kms of cropland/built-up land	Canada	[17]
	Land cover	Non-forested area	Urban and suburban areas are the main habitat	Connecticut, USA	[58]
	Climate	Salinity, pH, and temperature of water	Preferred larval environment is clean, sweet, slightly basic, warm water	Chile	[87]

Culex quinquefasciatus	Land cover	Urban areas	Human WNV disease incidence in Northeastern regions was positively associated with urban land covers	Northeastern USA	[57]
	Climate	Temperature	Female survival decreased significantly between 28 and 32C	Laboratory study	[42]
	Climate	Mean diurnal range	Negatively correlated	St Johns County, FL, USA	[65]
	Land cover	Leaf area index	Negatively correlated	St Johns County, FL, USA	[65]
	Land cover	Septic tanks	Septic tanks are important habitat	Puerto Rico	[88]
	Land cover	Container/urban areas	Containers/urban setting are an important habitat	Tampa, FL, USA	[59]
	Land cover	Sewage areas	Sewage areas were breeding sites	Atlanta, GA, USA	[89]
Culex restuans	Climate	Temperature	Female survival decreased significantly between 20 and 24C	Laboratory study	[42]
	Land cover	Urban and rural areas	Found in rural and urban settings	Iowa, USA	[55]
	Land cover				[90]
	Land cover	Residential areas	Higher number of mosquitoes in residential areas	New Jersey, USA	[91]
	Land cover	Urban areas	Egg raft rate was significantly higher in urban land use and land cover habitats.	Urbana-Champaign, IL, USA	[92]
	Land cover	High density canopy coverage	High-density canopy coverage was most frequently associated with high Culex abundance in oviposition traps.	Urbana-Champaign, IL, USA	[92]

	Land cover	Habitat quality and quantity	Female mosquitoes prefer nutrient-enriched containers and decrease ovipositing in containers with conspecific larvae	Southeastern MI, USA	[93]
	Land cover	Environmental correlates surrounding discarded tires	Culex were associated with factors related to the surrounding habitat (human population density, canopy cover, tire size)	Central IL, USA	[94]
Culex salinarius	Land cover	Areas with water (from NDVI)	Marshy land is the main habitat	Connecticut, USA	[58]
	Land cover	Salt marsh	Larvae found in undisturbed salt marshes	Suffolk Co, NY, USA	[95]
	Land cover	Freshwater wetlands	Larvae not considerably found	Suffolk Co, NY, USA	[95]
	Land cover	Artificial containers	Larvae not considerably found	Suffolk Co, NY, USA	[95]
	Land cover	Salt marshes	Adults found significantly more than at upland sites	Suffolk Co, NY, USA	[95]
	Climate	Season	Females most prevalent in spring and fall but active year-round	Chambers CO, TX, USA	[96]
	Climate	Ambient temperature	No effect	Iowa, USA	[97]
	Climate	Relative humidity	No effect	Iowa, USA	[97]
	Land cover	Shade	More commonly in deep shade	Delaware, USA	[98]
	Land cover	Wetland type	Mostly at Conservation Enhancement and Preservation Program ponds vs. retention ponds and constructed wetlands	Delaware, USA	[98]
	Land cover	Vegetation	More abundant in ponds with loosestrife, grasses (Poaceae), duckweed, and Phragmites, and tend to be	Delaware, USA	[98]

			found in permanent ponds or wetlands		
	Topography	Vegetation	Attracted to ground-based light traps than traps suspended in the tree canopy	Connecticut, USA	[99]
	Climate	Temperature	Prefers cooler/spring in FL	Florida, USA	[100]
Culex tarsalis	Land cover	Agricultural areas	Human WNV disease incidence in the western US was positively associated with agricultural land covers.	Western USA	[57]
	Land cover	Vegetation	Positively correlated with grass/hay	Sioux Falls, SD, USA	[101]
	Land cover	Rural areas	Found in rural settings	Iowa, USA	[55]
	Land cover	Grassland/prairie cover	Grassland cover is favorable habitat for the vector	Alberta, Manitoba, and Saskatchewan, Canada	[16]
	Climate	Monthly temperatures	Temperature is important habitat consideration	Alberta, Manitoba, and Saskatchewan, Canada	[16]
	Climate	Monthly precipitation	Increases with vector abundance; negatively coefficient for WNV infection rate model	Alberta, Manitoba, and Saskatchewan, Canada	[16]
	Climate	Future climate scenarios	Vector abundance and WNV infection rate increase with a warm & dry climate	Alberta, Manitoba, and Saskatchewan, Canada	[16]
	Land cover	Irrigated land area	Positively correlated with vector abundance; irrigated land may make good habitat for vector	North-central CO, USA	[102]
	Topography	Elevation	Negatively correlated with vector abundance	North-central CO, USA	[102]
	Land cover	Impervious surface	Negatively correlated with vector abundance, but a positive regression coefficient	North-central CO, USA	[102]

Climate	Dew point	Positively correlated with vector abundance	Bismark, ND, USA	[103]
Climate	Day length	Positively correlated with vector abundance	Bismark, ND, USA	[103]
Climate	Number of days below 0ºC	Positively correlated with abundance	Bismark, ND, USA	[103]
Land cover	Flood gauge height	Negatively correlated with vector abundance due to extreme flooding	Bismark, ND, USA	[103]

Table S4. Percent environmental variable contribution during Maxent model development for each *Culex* species.

Category	Environmental variable	*Pipiens*	*Restuans*	*Salinarius*	*Tarsalis*	*Erraticus*	*Nigripalpus*	*Quinquefasciatus*
Climate	Annual mean temperature	14.7	0.2	0.6	21.4	0.0	0.9	18.2
	Temperature annual range	0.1	0.7	0.0	0.0	0.1	0.0	0.2
	Mean diurnal temp. range	2.3	0.9	0.0	0.0	0.0	0.0	2.3
	Max. temp. in the warmest month	0.0	6.6	0.0	8.9	0.0	0.1	2.7
	Min. temp. in the coldest month	1.6	1.1	0.4	2.6	11.8	0.3	13.8
	Annual mean specific humidity	1.8	0.7	8.3	0.8	0.0	0.5	0.0
	Specific humidity in the most humid month	6.4	0.4	5.8	14.1	15.7	71.7	1.3
	Specific humidity in the least humid month	0.4	1.6	0.0	1.9	0.6	2.0	0.4
Land cover	Evergreen/deciduous needleleaf trees	<0.1	17.5	0.2	0.0	0.0	4.3	0.6
	Evergreen broadleaf trees	0.0	0.4	0.0	0.0	0.5	1.2	8.7
	Deciduous broadleaf trees	0.5	1.0	0.0	0.8	0.3	0.3	0.1
	Mixed/other trees	0.3	1.0	0.0	0.8	2.3	0.2	2.0
	Shrubs	0.0	19.2	4.9	0.1	3.7	0.3	5.3
	Herbaceous vegetation	0.0	15.7	0.3	0.3	3.8	1.2	1.8
	Cultivated and managed vegetation	8.8	2.9	34.1	35.8	36.7	5.3	4.0
	Regularly flooded vegetation	0.0	2.4	0.0	0.0	0.0	0.1	0.0
	Urban/built-up	57.4	11.7	33.8	12.0	13.3	1.8	32.9
	Snow/ice	0.0	0.0	0.0	0.3	0.0	0.0	0.0
	Barren	0.0	0.0	0.0	0.0	0.0	0.0	0.1
	Open water	2.7	2.7	0.2	0.0	1.4	1.2	1.3
Habitat	Evenness of EVI	0.0	3.4	0.0	0.0	0.7	1.2	0.1
Topography	Elevation	2.9	6.1	5.1	0.2	9.1	2.5	3.6
	Roughness index	0.0	0.4	0.3	0.0	0.1	0.5	0.0
	Slope	0.0	2.8	2.3	0.0	0.1	3.6	0.5
	Terrain ruggedness index	0.0	0.6	3.7	0.0	0.0	0.8	0.1

Figures:

Figure S1. Maps of the environmental training area unique to each species used for the Maxent models across North America for (a) *Cx. pipiens*, (b) *Cx. restuans*, (c) *Cx. salinarius*, and (d) *Cx. tarsalis*. These were created by buffering the data based on the median distance from each presence data point to the centroid of all presence points. Ten thousand background points are randomly sampled from the shaded environmental training area when running Maxent.

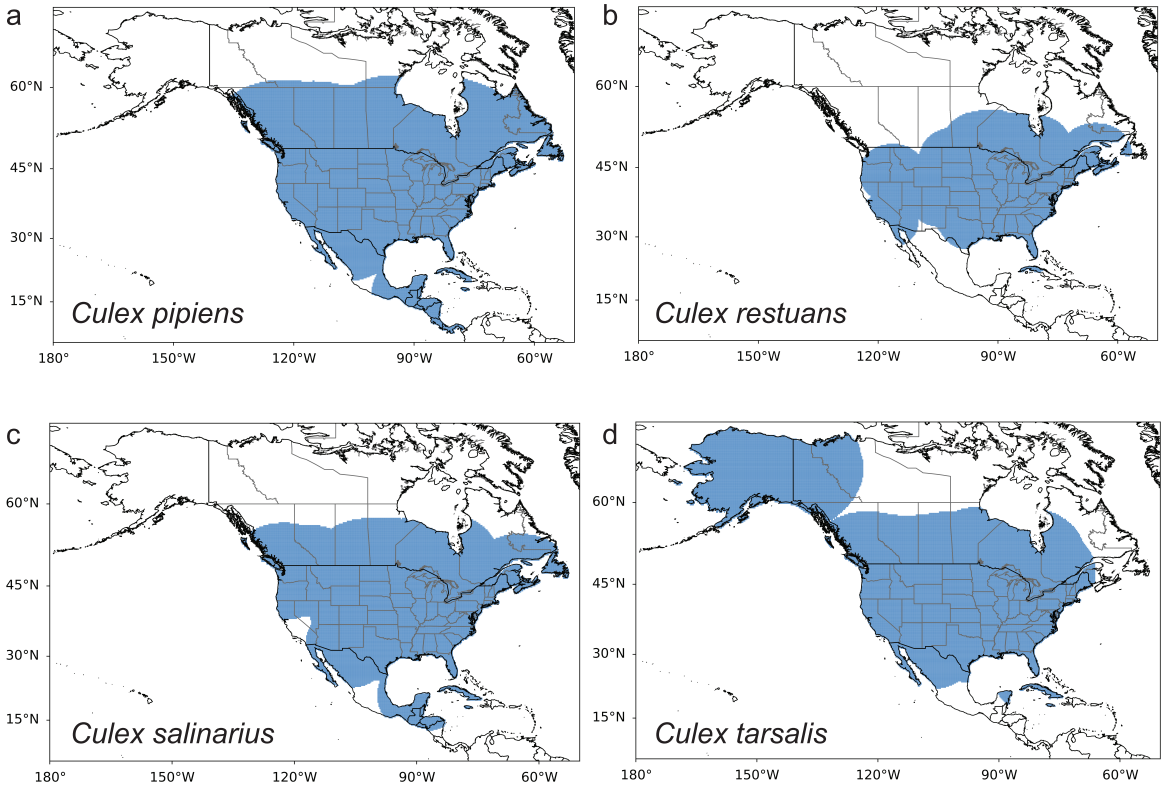

Figure S2. Maps of the environmental training area unique to each species used for the Maxent models across North and South America for (a) *Cx. erraticus*, (b) *Cx. nigripalpus*, and (c) *Cx. quinquefasciatus*. These were created by buffering the data based on the median distance from each presence data point to the centroid of all presence points. Ten thousand background points are randomly sampled from the shaded environmental training area when running Maxent.

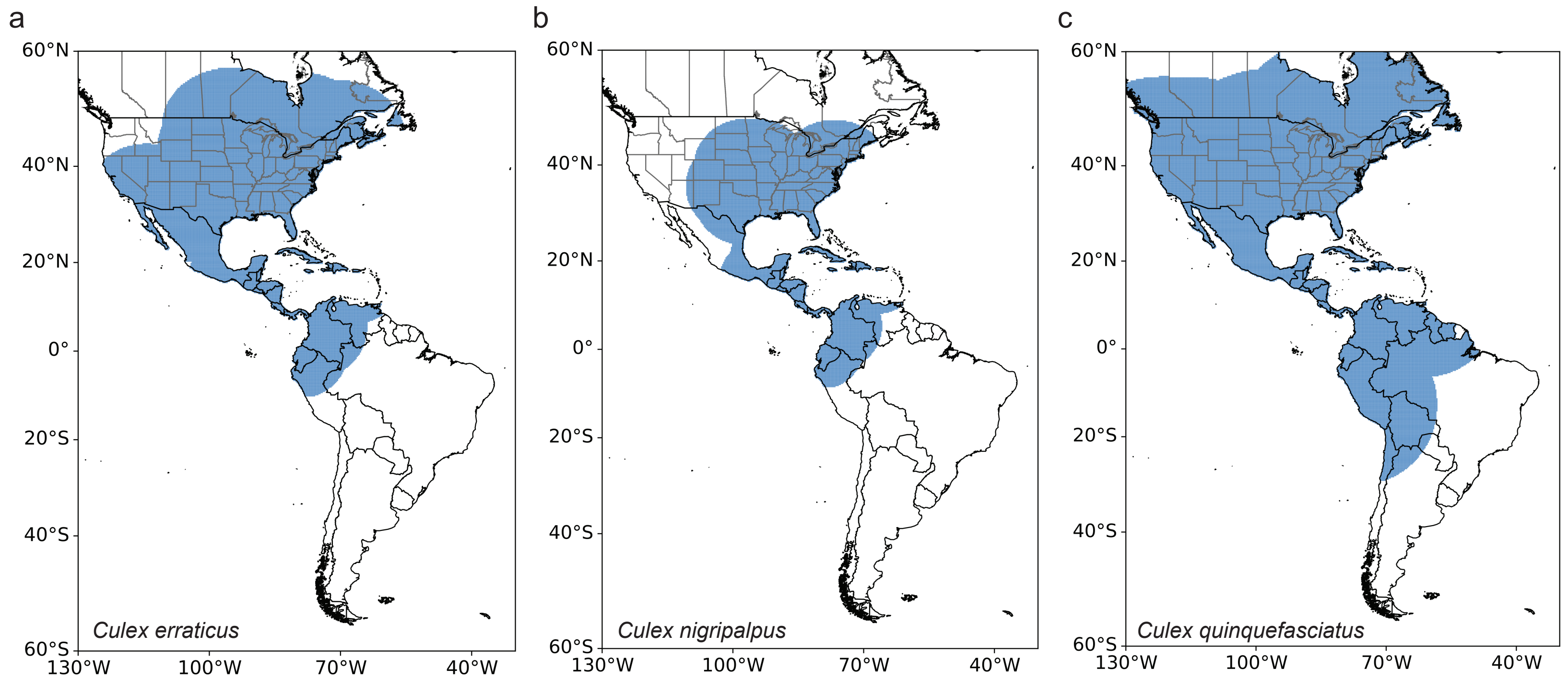

Figure S3. After extrapolating the Maxent models across North America, areas that are highlighted have novel climate or environmental conditions relative to the background environmental training dataset. This is unique to each species: (a) *Cx. pipiens*, (b) *Cx. restuans*, (c) *Cx. salinarius*, and (d) *Cx. tarsalis*.

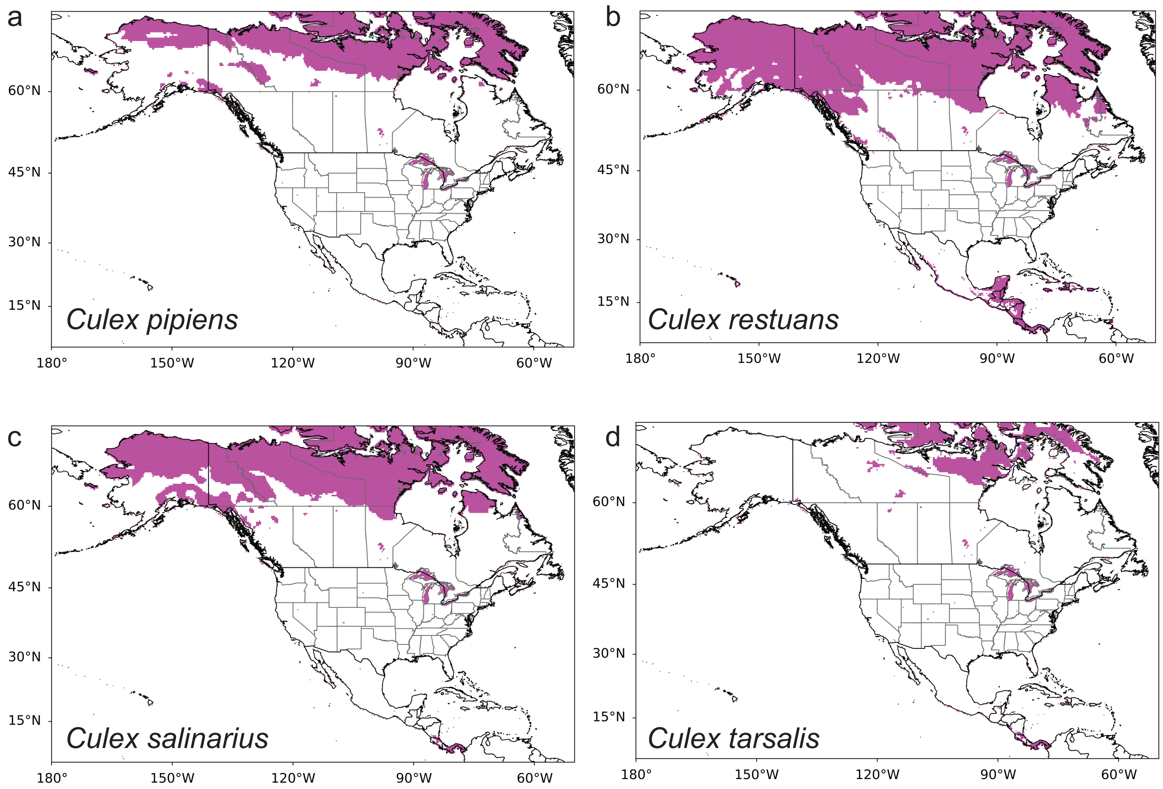

Figure S4. After extrapolating the Maxent models across North and South America, areas that are highlighted have novel climate or environmental conditions relative to the background environmental training dataset. This is unique to each species: (a) *Cx. erraticus*, (b) *Cx. nigripalpus*, and (c) *Cx. quinquefasciatus*.

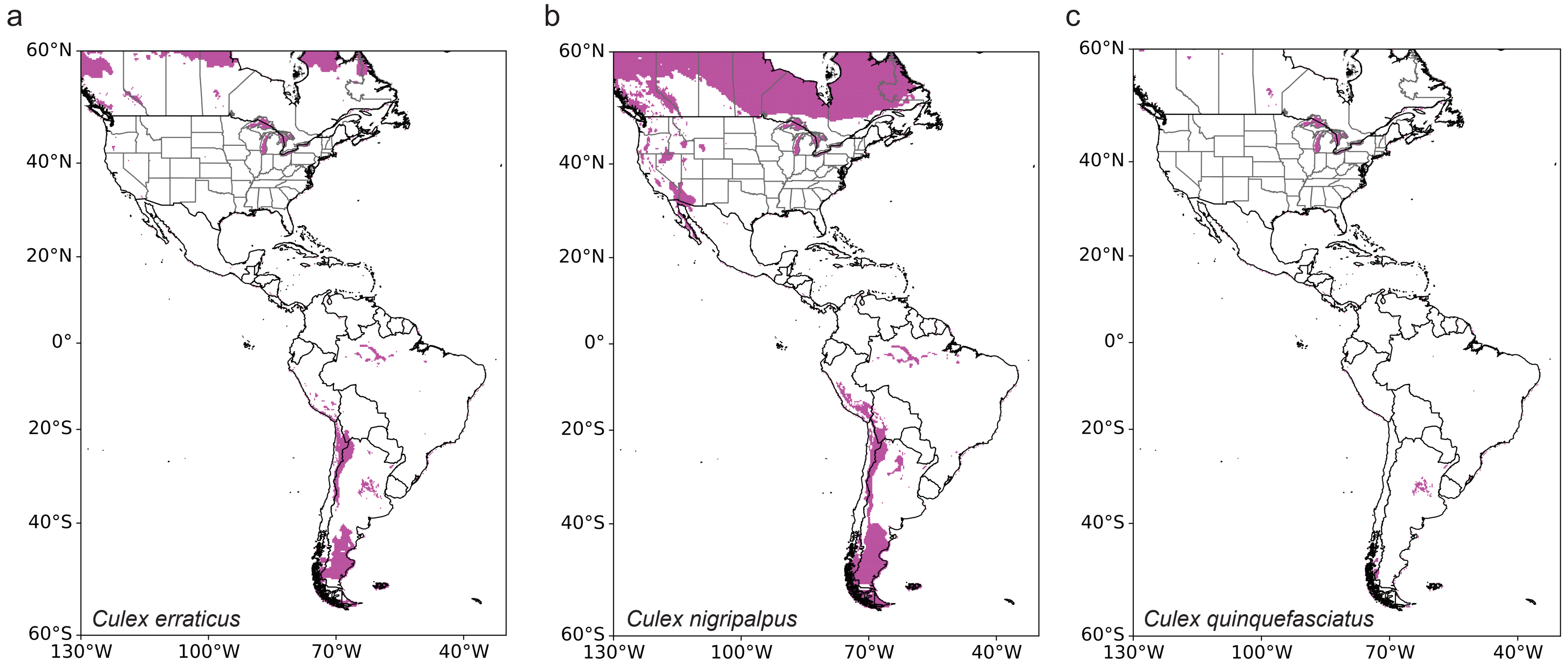

Figure S5. Maps of the difference between the maximum and minimum suitability output amongst the ten bootstrapped replicates (i.e., the range) to show areas of high or low uncertainty in our models for species in North America: (a) *Cx. pipiens*, (b) *Cx. restuans*, (c) *Cx. salinarius*, and (d) *Cx. tarsalis*.

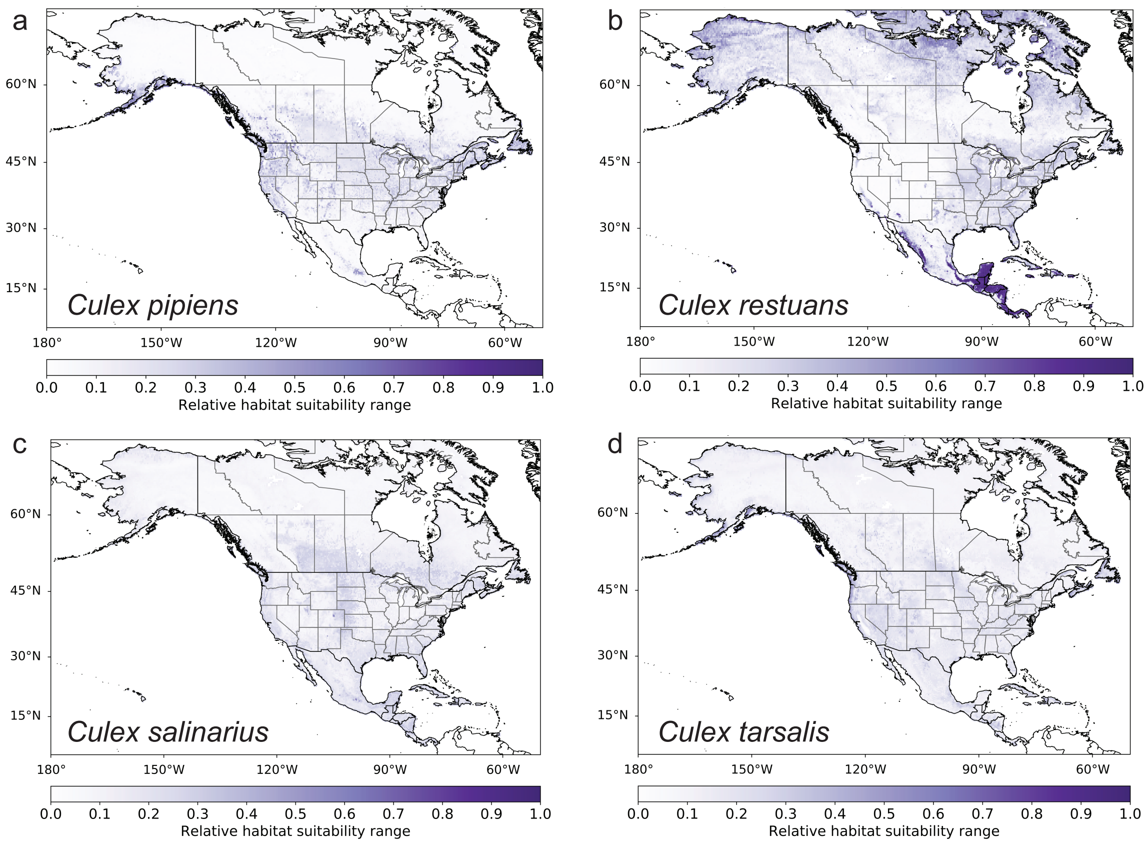

Figure S6. Maps of the difference between the maximum and minimum suitability output amongst the ten bootstrapped replicates (i.e., the range) to show areas of high or low uncertainty in our models for species in North and South America: (a) *Cx. erraticus*, (b) *Cx. nigripalpus*, and (c) *Cx. quinquefasciatus*.

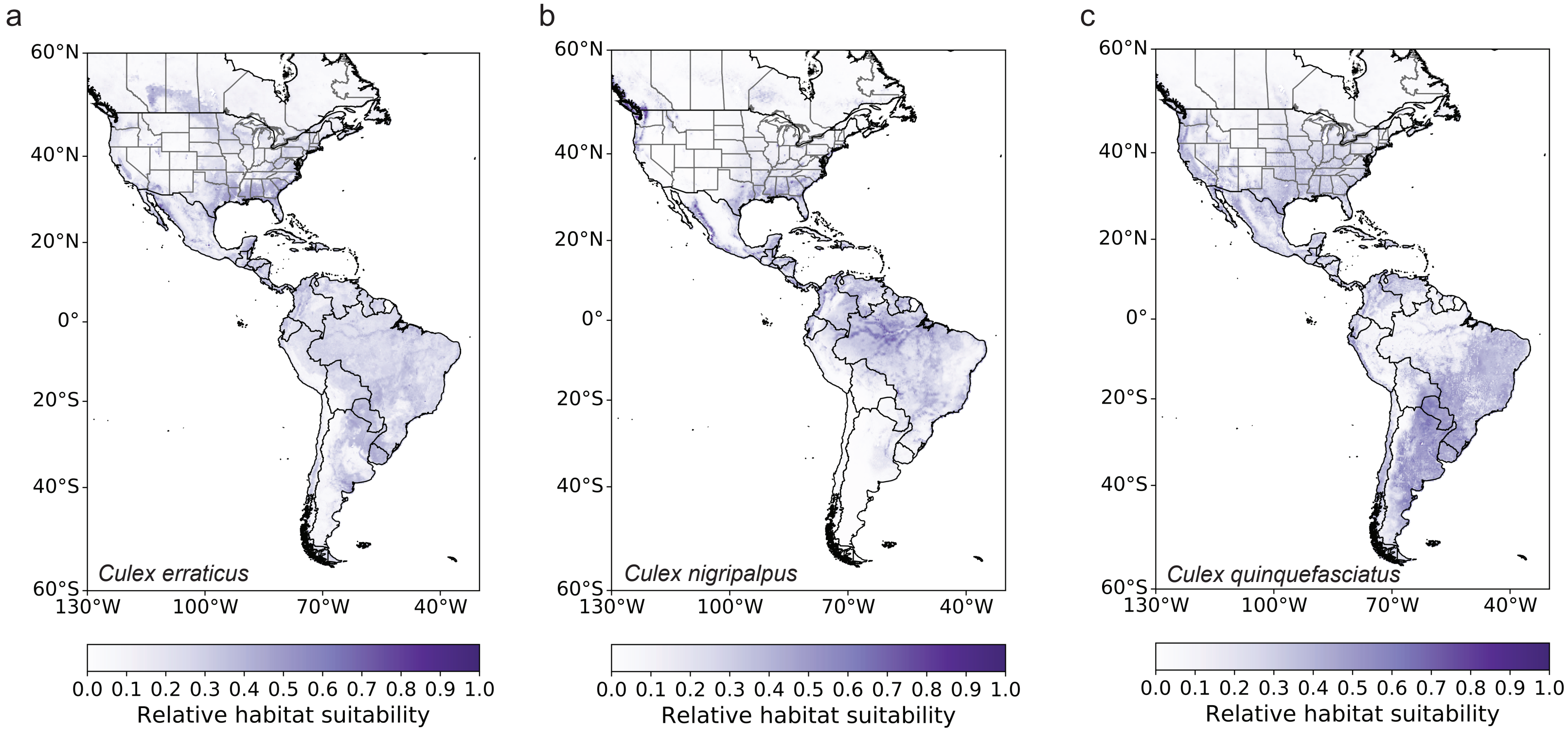

Supplement: Supplementary file 1 — Additional file 1: Table S1. The total number of presence data points for each Culex species used in model development after filtering by the 30-km radial buffer. Table S2. Final model specifications and performance metrics for each species. Table S3. Summary of environmental factors important for each of the seven Culex species, which were obtained from the literature review. Table S4. Percent environmental variable contribution during Maxent model development for each Culex species. Figure S1. Maps of the environmental training area unique to each species used for the Maxent models across North America for a Culex pipiens, b Culex restuans, c Culex salinarius, and d Culex tarsalis. These were created by buffering the data based on the median distance from each presence data point to the centroid of all presence points. Ten thousand background points are randomly sampled from the shaded environmental training area when running Maxent. Figure S2. Maps of the environmental training area unique to each species used for the Maxent models across North America and South America for a Culex erraticus, b Culex nigripalpus, and c Culex quinquefasciatus. These were created by buffering the data based on the median distance from each presence data point to the centroid of all presence points. Ten thousand background points are randomly sampled from the shaded environmental training area when running Maxent. Figure S3. After extrapolating the Maxent models across North America, areas that are highlighted have novel climate or environmental conditions relative to the background environmental training dataset. This is unique to each species: a Culex pipiens, b Culex restuans, c Culex salinarius, and d Culex tarsalis. Figure S4. After extrapolating the Maxent models across North America and South America, areas that are highlighted have novel climate or environmental conditions relative to the background environmental training dataset. This is unique to each species: a Culex erra [file 13071_2021_5051_MOESM1_ESM.pdf]
